# Comparison of ^18^F-FDG-PET and ^18^F-FDG-PET/CT for the diagnostic performance in thyroid nodules with indeterminate cytology: A meta-analysis

**DOI:** 10.1097/MD.0000000000020446

**Published:** 2020-05-29

**Authors:** Wan Qichang, Shen Jinming, Li Lu, Ji Bin, Wang Renjie, Zheng Xiuying

**Affiliations:** a Department of Nuclear Medicine, China-Japan Union Hospital of Jilin University, Changchun, China; b Department of Intensive Care, Xinyu People's Hospital, Xinyu, China; c Department of Discipline Inspection, The Second Affiliated Hospital of Mudanjiang Medical University, Mudanjiang, China.

**Keywords:** diagnostic performance, FDG-PET, FDG-PET/CT, thyroid nodules with indeterminate cytology

## Abstract

**Backgrounds:**

We performed a meta-analysis to compare ^18^F-FDG-PET and ^18^F-FDG-PET/CT for the diagnostic performance in thyroid nodules with indeterminate cytology by Bethesda classification.

**Methods:**

PubMed and Embase databases were searched for eligible studies from the earliest available date of indexing through September 2019. Only studies with clearly stated histopathology confirmation were included. Publication bias was assessed by Deeks funnel plot. The pooled sensitivity, specificity, positive likelihood ratio, negative likelihood ratio, positive predictive value, negative predictive value, and diagnostic odds ratio was determined by random-effect analysis, respectively. All diagnostic estimate indexes were determined separately for PET alone and PET/CT and were compared pair-wisely using *Z*-test.

**Results:**

We included 214 patients from five PET alone studies and 420 patients from 8 PET/CT studies in this meta-analysis. The range of the prevalence of malignancy was 11% to 27% for PET alone studies (Median, 20%) and 4% to 50% for PET/CT studies (Median, 24%). The sensitivity (0.95 vs 0.73, *P* *<* .01), negative likelihood ratio (0.20 vs 0.53, *P =* .04) and negative predictive value (0.99 vs 0.91, *P* *<* .01) of PET alone are significantly better than those of PET/CT. For PET/CT, Fagan nomogram indicated that when the pre-test probability was set at 24%, the negative post-test probability could decrease to 12%.

**Conclusion:**

This meta-analysis reveals that in evaluating thyroid nodules with indeterminate cytology by Bethesda classification, the rule-out performance of ^18^F-FDG-PET is significantly better than ^18^F-FDG-PET/CT, although the latter represents a more objective and accurate technique. We hypothesize that the lack of precise localization of suspicious FDG uptake in the neck region may have contributed to this overvaluation for PET alone studies, and advocate that future studies be performed with PET/CT rather than PET alone to avoid misinterpretation and overvaluation in this scenario.

## Abstract 2

Supplemental Digital Content is available in the text

## 1 Introduction

High rates of thyroid nodules with indeterminate cytology (TNIC), which belong to the Bethesda category III or IV, have subjected more patients to diagnostic surgeries.^[1,2]^ However, because these surgeries could cause significant emotional stress, economic burden and risk of surgical complications among patients with ultimate benign nodules, which account for 70% to 85% of the total cases, it is well accepted that a large number of them are unnecessary.^[3,4]^ At present, there is a need for well-validated tools to further evaluate the risk of these nodules before surgical procedures are resorted to.

During the past decades, many studies have reported that ^18^F-FDG-PET and ^18^F-FDG-PET/CT have high sensitivity and high negative predictive value (NPV) in evaluating TNIC.^[5–16]^ However, these have not been reflected in the current American Thyroid Association (ATA) guidelines due to co-existent variable and sometimes conflicting results in the literature.^[17]^ For example, in a most recent meta-analysis which exclusively analyzed PET/CT studies, Castellana et al reported only moderate sensitivity and NPV in this clinical setting.^[18]^

PET and PET/CT are both molecular imaging techniques that could provide functional or metabolic information of the diseases. Nevertheless, a major disadvantage of PET is the lack of anatomical information to precisely localize any suspicious tracer accumulation.^[19]^ PET/CT, by simultaneously fusing the PET data and the CT data, could overcome this limitation. For this reason, in evaluating TNIC, the diagnostic performance of PET/CT might be different from PET alone. However, as far as we know, few studies have evaluated the relative effectiveness of these 2 techniques. Therefore, by summarizing the current evidence in the literature, we conducted a meta-analysis to compare ^18^F-FDG-PET and ^18^F-FDG-PET/CT for the diagnostic performance in TNIC by Bethesda classification.

## 2 Materials and methods

This study was conducted according to the Preferred Reporting Items for Systematic Reviews and Meta-Analyses guidelines ^[20]^ and specific suggestions for meta-analyses of studies investigating diagnostic accuracy were followed.^[21]^

### 2.1 Data sources and search strategy

Two authors searched all available literature published until September 2019 in the PubMed and Embase database using an algorithm based on a combination of terms:

1. “Fluorodeoxyglucose F18” [Mesh] OR FDG OR ∗deoxygluco∗ OR F-DG OR fluorodeoxygluc∗
2. “positron emission tomography” [Mesh] OR PET OR PET/CT OR “positron emission tomography/computed tomography”
3. “thyroid nodule” [Mesh] OR “thyroid nodule∗”
4. indeterminat∗ OR TIR3 OR TIR3A OR TIR3B OR Thy3a OR Thy3f OR AUS OR FLUS OR “atypia of undetermined significance” OR “follicular lesion of undetermined significance” OR “Follicular neoplasm” OR “Follicular adenoma∗” OR “Hürthle cell neoplasm” OR “Hürthle cell adenoma∗.”

Additional key words identified during the searching process were incorporated into the searching strategies. The reference lists of identified publications were also hand-searched for potentially relevant studies.

## 3 Study selection

Studies were eligible for inclusion if all of the following criteria applied:

1. the diagnostic performance of ^18^F-FDG-PET or ^18^F-FDG-PET/CT in patients with thyroid nodule of indeterminate cytology (Bethesda category III and IV) could be clearly identified in the study or subsets of the study;
2. the reference standard was histopathology confirmation and was clearly stated in the article;
3. the data were sufficient (ie, patient number above 10) to construct a 2 × 2 contingency table.

The exclusion criteria were:

1. duplicated articles;
2. case reports, abstract, letters, review or meta-analyses;
3. clearly irrelevant titles and abstracts;
4. non-English full–text articles.

Using the afore-mentioned inclusion and exclusion criteria, three researchers independently screened titles and abstracts of the retrieved articles and then evaluated the full-text version of the remaining articles to determine their eligibility for inclusion. Disagreements between the researchers were resolved by discussion, or if necessary, by a fourth party.

### 3.1 Quality assessment

Two reviewers independently assessed the quality of the included studies based on the Quality Assessment of Diagnostic Accuracy Studies (QUADAS-2) tool.^[22]^ Each study was evaluated based on the following domains: patient selection, index test, reference standard and, flow and timing. These domains were then evaluated according to the risk of bias and were rated regarding applicability as ‘high’, ‘low’ or ‘unclear’. Disagreements between the reviewers were resolved by discussion, or if necessary, by a third party.

### 3.2 Data extraction

Two reviewers independently conducted data extraction for all included articles. The extracted data included the first author, year, study characteristics (country, data style, study design, image analysis, device and definition of positivity), patient characteristics (numbers of patients, female percentage, mean age), and nodule characteristics (nodule size, source of size data, FNA results and histopathology of malignant nodules). Disagreements between the reviewers were resolved by discussion, or if necessary, by a third party. For each study, the absolute numbers of true-positive, false-positive, false-negative and true-negative data were also extracted.

### 3.3 Statistical analysis

The pooled sensitivity, specificity, positive likelihood ratio (PLR), negative likelihood ratio (NLR), positive predictive value (PPV), NPV and diagnostic odds ratio (DOR) for ^18^F-FDG-PET and ^18^F-FDG-PET/CT in diagnosing TNIC by Bethesda classification were presented as estimates with 95% confidence intervals (CIs) by using random-effect analysis. Heterogeneity among pooled studies was assessed by use of Cochrane *Q* test and *I*^2^ statistic. Values of *I*^2^ equal to 25%, 50%, and 75% were assumed to represent low, moderate, and high heterogeneity, respectively. In case of substantial heterogeneity, meta-regression analysis was performed to explore potential source of heterogeneity and the covariates were:

1. study design (prospective vs retrospective);
2. image analysis (visual vs quantitative),
3. data style (patient-based vs lesion-based);
4. sample size (≥50 vs <50).

Publication bias was assessed by Deeks funnel plot. A Fagan nomogram was used to visualize the association between pre-test and post-test probability according to the calculated likelihood ratio of the test.

The pooled sensitivity, specificity, PLR, NLR, and DOR were analyzed by using Meta-disc (Meta-DiSc, version 1.4, Universidad Complutense, 189 Madrid). The pooled PPV and NPV were analyzed by using bivariate logitnormal random-effects model in R software.^[23]^ The nomogram was plotted by using midas package in Stata 15.1 (Stata Corporation, College Station, TX).^[24]^

### 3.4 Ethical approval

This is a meta-analysis involving data which were extracted from previously published original studies. Therefore, no ethical approval was required since the authors were not involved in carrying out experiments on animals or humans for this analysis.

## 4 Results

### 4.1 Literature search and study selection

**Figure 1:**
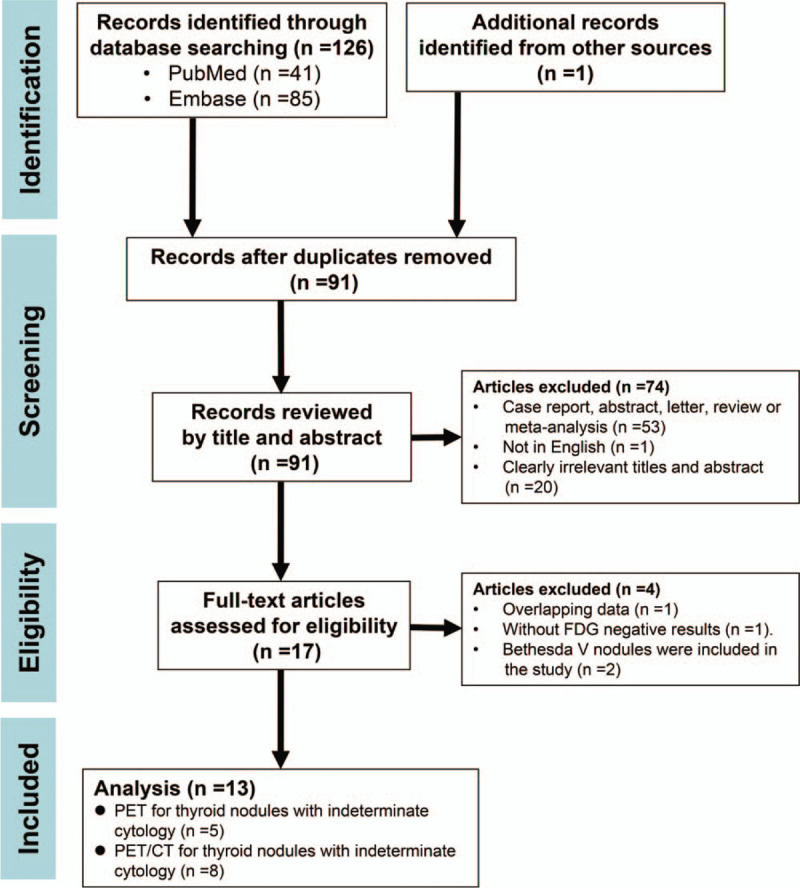
The Preferred Reporting Items for Systematic Reviews and Meta-Analyses flow diagram of study selection.

The initial search retrieved 127 articles, and 110 were excluded upon review of titles and abstracts. The remaining 17 articles were carefully assessed by full-text and another 4 were excluded for the following reasons: overlapping data (n = 1); without FDG negative results (n = 1); Bethesda V nodules were included in the study (n = 2). Finally, 13 articles^[5–14,25–27]^ including data on the diagnostic performance of ^18^F-FDG-PET or ^18^F-FDG-PET/CT in TNIC by Bethesda classification were eligible for further analysis. A Preferred Reporting Items for Systematic Reviews and Meta-Analyses flow diagram of the study selection process is shown in Figure 1.

### 4.2 Study description and quality assessment

The first author, study characteristics and patient characteristics of the 13 articles comprising 634 patients are presented in Table 1. The nodule characteristics are shown in Table 2.

**Table 1:**
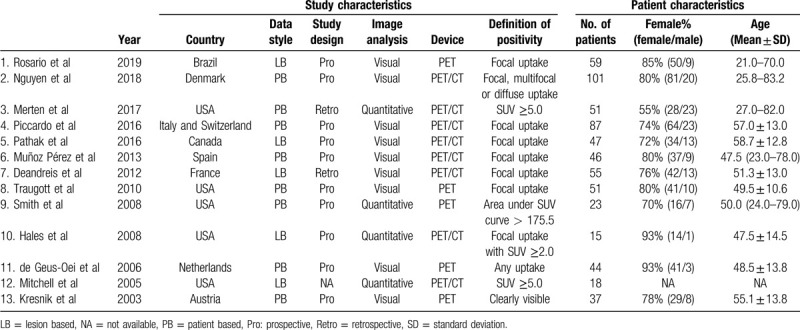
Study and patient characteristics of the included studies.

**Table 2:**
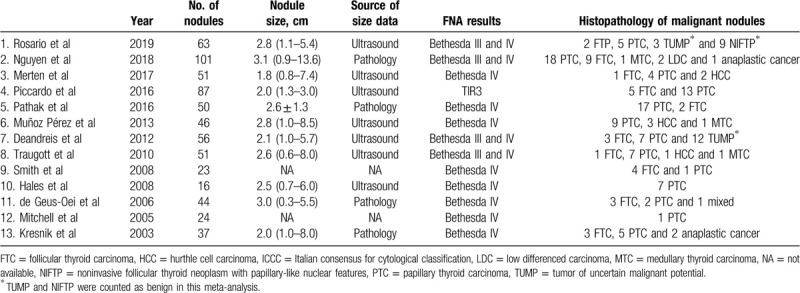
Nodule characteristic of the included studies.

**Figure 2:**
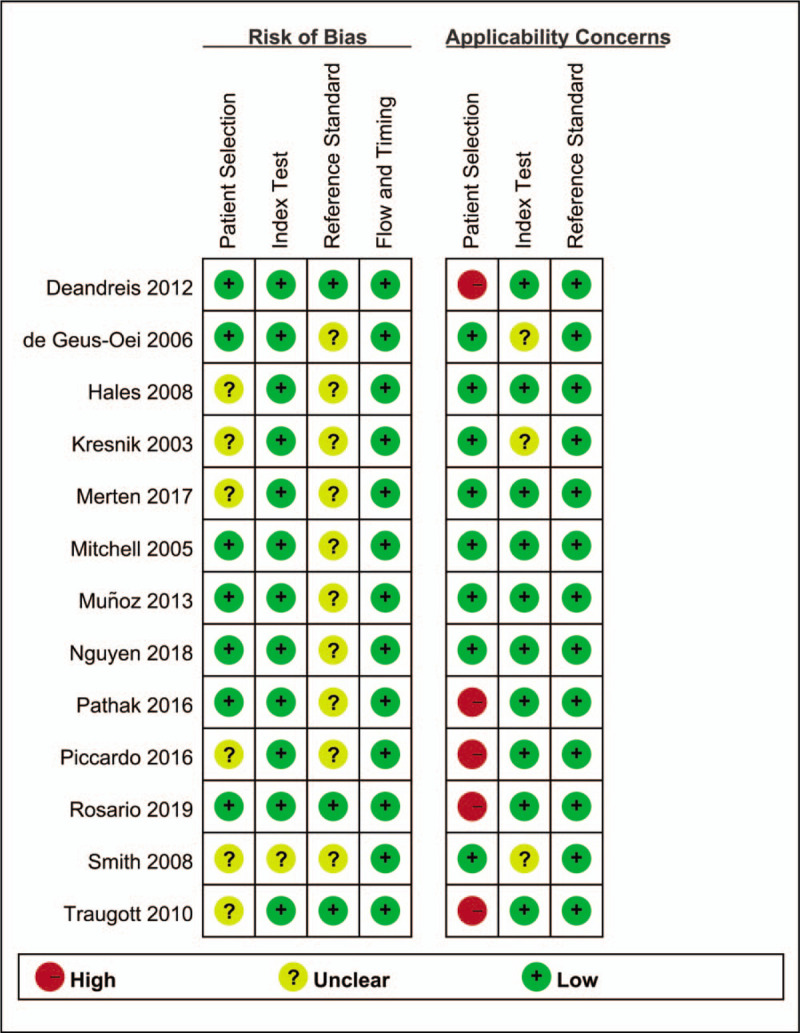
Summary of the quality assessment of each study.

The results of summary risk of bias and applicability concerns of each study are shown in Figure 2. The quality of the included studies was considered satisfactory.

### 4.3 Diagnostic performance of ^18^F-FDG-PET in TNIC by Bethesda classification

For ^18^F-FDG-PET, the diagnostic performance was reported in 5 studies comprising 214 patients. The range of the prevalence of malignancy for the included studies was 11% to 27%, and the median was 20%.

**Figure 3:**
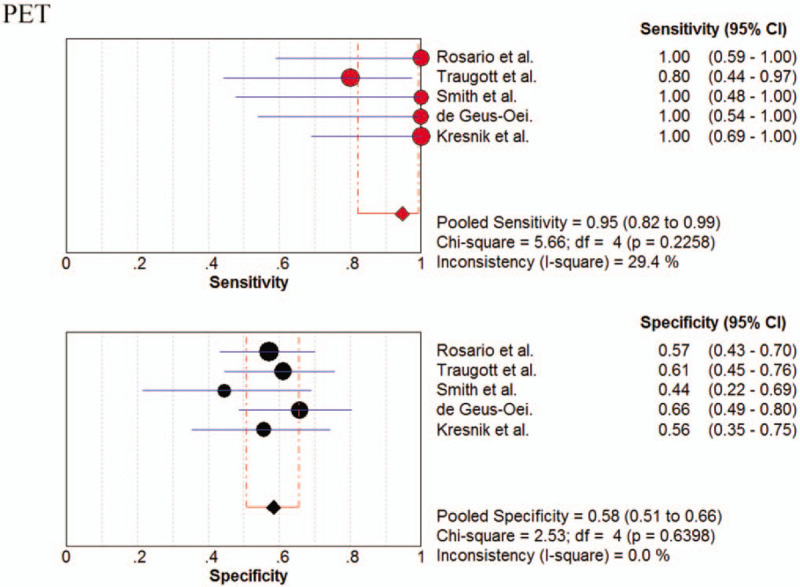
Forest plot of pooled sensitivity and specificity for ^18^F-FDG-PET in diagnosing thyroid nodules with indeterminate cytology by Bethesda classification.

The pooled sensitivity, specificity, PLR, NLR, PPV, NPV, and DOR were 0.95 (95% CI; 0.82–0.99), 0.58 (95% CI; 0.51–0.66), 2.13 (95% CI; 1.75–2.58), 0.20 (95% CI; 0.08–0.51), 0.34 (95% CI; 0.26–0.45), 0.99 (95% CI; 0.96–1.00), and 11.51 (95% CI; 3.80–34.84), respectively. Figure 3 shows the forest plots of the sensitivity and specificity for ^18^F-FDG-PET in diagnosing TNIC. The I^2^ values for sensitivity and specificity were 29% and 0%, respectively. No publication bias was found (*P* = .94).

### 4.4 Diagnostic performance of ^18^F-FDG-PET/CT in TNIC by Bethesda classification

For ^18^F-FDG-PET/CT, the diagnostic performance was reported in 8 studies comprising 420 patients. The range of the prevalence of malignancy for the included studies was 4% to 50%, and the median was 24%.

**Figure 4:**
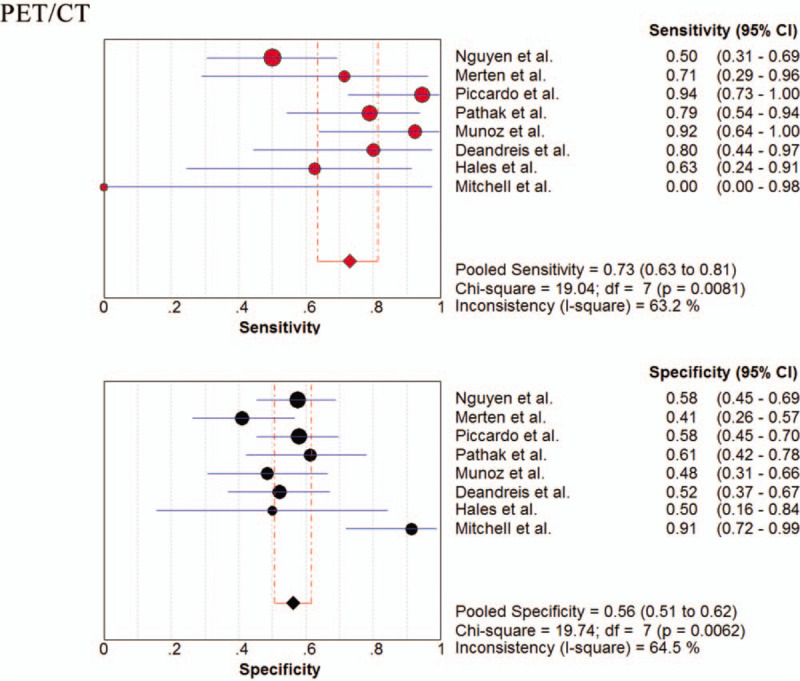
Forest plot of pooled sensitivity and specificity for ^18^F-FDG-PET/CT in diagnosing thyroid nodules with indeterminate cytology by Bethesda classification.

The pooled sensitivity, specificity, PLR, NLR, PPV, NPV, and DOR were 0.73 (95% CI; 0.64–0.81), 0.56 (95% CI; 0.51–0.62), 1.70 (95% CI; 1.40–2.06), 0.53 (95% CI; 0.31–0.89), 0.37 (95% CI; 0.29–0.47), 0.91 (95% CI; 0.85–0.97), and 3.51 (95% CI; 1.68–7.34), respectively. Figure 4 shows the forest plots of the sensitivity and specificity for ^18^F-FDG-PET/CT in diagnosing TNIC. The I^2^ values for sensitivity and specificity were 63% and 65%, respectively. No publication bias was found (*P* = .88).

Because heterogeneity between studies was high for sensitivity, with no evidence of a threshold effect (ρ = 0.238, *P* = .570), meta-regression analysis was performed to explore other sources of heterogeneity, but we did not identify the source of heterogeneity (Supplement Table 1, http://links.lww.com/MD/E309).

### 4.5 Comparison of ^18^F-FDG-PET and ^18^F-FDG-PET/CT in diagnosing TNIC by Bethesda classification

Pair-wise comparisons for all the diagnostic estimate indexes were shown in Table 3. We found that the sensitivity (0.95 vs 0.73, *P* *<* .01), NLR (0.20 vs 0.53, *P* *=* .04) and NPV (0.99 vs 0.91, *P* *<* .01) of ^18^F-FDG-PET were significantly better than those of ^18^F-FDG-PET/CT in diagnosing TNIC by Bethesda classification.

**Table 3:**
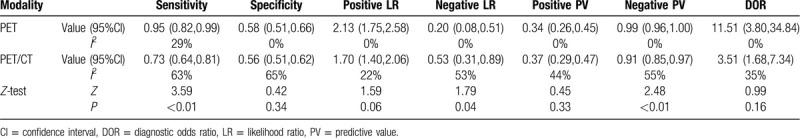
Pair-wise comparison for diagnostic estimates between PET and PET/CT.

### 4.6 Fagan nomogram

**Figure 5:**
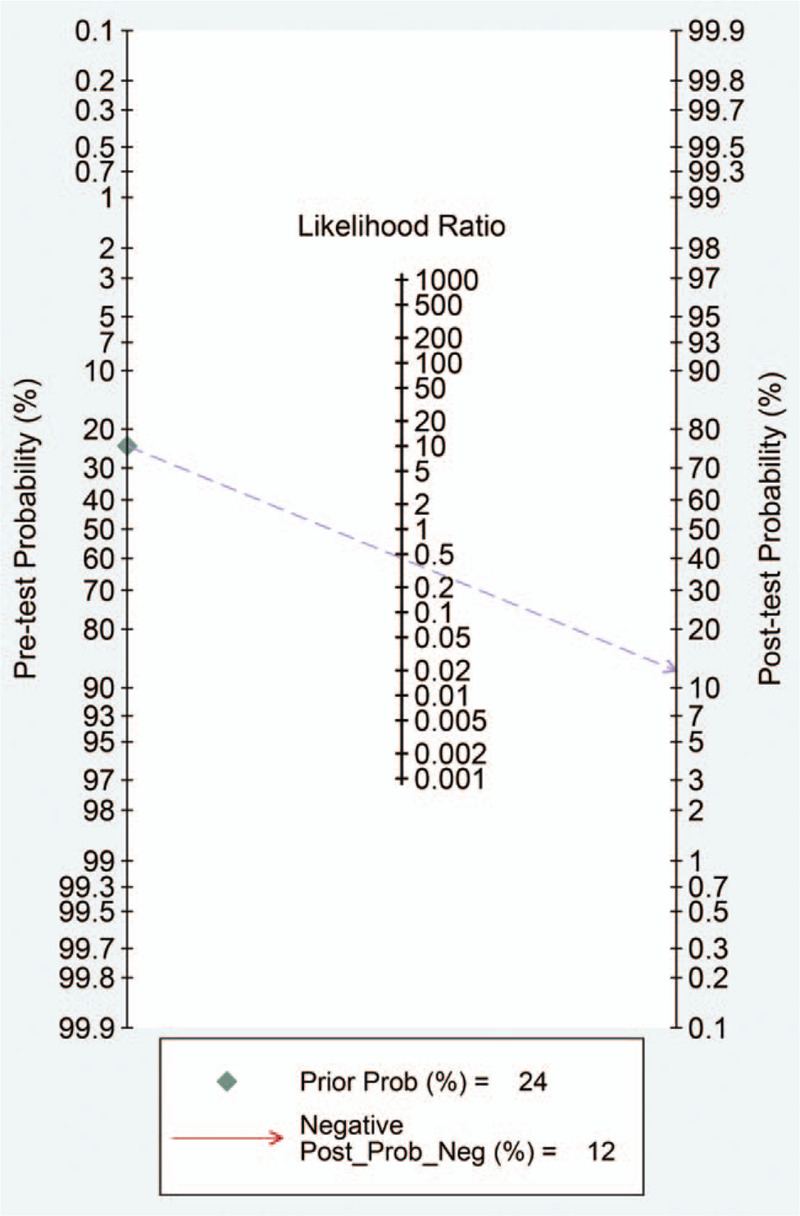
Fagan nomogram of pre-test probability and post-test probability for ^18^F-FDG-PET/CT studies. The pre-test probability was set at 24%.

Fagan nomogram indicated that when the pre-test probability was set at 24%, which is the median value derived from PET/CT studies, the negative post-test probability (risk of malignancy upon a negative PET/CT result) could decrease to 12% (Fig. 5).

## 5 Discussion

We demonstrated that in evaluating TNIC by Bethesda classification, the sensitivity (0.95 vs 0.73, *P* *<* .01), NLR (0.29 vs 0.53, *P* *=* .04) and NPV (0.99 vs 0.91, *P* *<* .01) of ^18^F-FDG-PET are significantly better than those of ^18^F-FDG-PET/CT. However, for all the PET/CT studies that were included in this meta-analysis, the definition of positive cases was solely based on the pattern and degree of the FDG uptake. In other words, rather than providing additional diagnostic information, the CT part in PET/CT was only used to confirm or correct the anatomical location of suspicious FDG uptake. Additionally, dedicated PET alone scanners, which is still in use to evaluate TNIC by several researchers,^[10]^ have poorer resolution compared with the modern day hybrid PET/CT systems. Taken together, PET/CT should be more objective and accurate than PET alone in terms of diagnostic purpose. The resulting “superiority” of PET alone over PET/CT in this study was not reasonable. Thus, it can be inferred that the rule-out performance of ^18^F-FDG-PET in TNIC by Bethesda classification might be significantly overvalued in the literature.

Several previous meta-analyses have described the diagnostic performance of ^18^F-FDG-PET and/or ^18^F-FDG-PET/CT in evaluating TNIC. In 2011, Vriens et al included 5 PET alone studies and one PET/CT studies and reported a sensitivity of 0.95 and an NPV of 0.96.^[15]^ In 2013, Wang et al analyzed four PET alone studies and 1 PET/CT study and yielded a sensitivity of 0.89.^[16]^ Most recently, Castellana et al exclusively analyzed 8 PET/CT studies and reported a sensitivity of 0.74 and an NPV of 0.74.^[15,16,18]^ It can be seen that the rule-out performance becomes lower as the number of included PET alone studies decreases. The significant differences between PET and PET/CT in rule-out performance might be able to help explain the variable results in these meta-analyses over the years.

For the reasons as to why the rule-out performance of ^18^F-FDG-PET in TNIC by Bethesda classification was overvalued, apart from the small patient number and possible publication bias (although we did not find publication bias using Deeks funnel plot for PET alone studies), one speculation is that the atypical FDG uptakes in the neck region might have played an important role. Because the neck region are highly complicated anatomical districts and are characterized by various physiological or pathological tracer uptakes (eg, by the tonsils, pharynx, cervical lymph nodes, and other thyroid nodules),^[28,29]^ these atypical tracer uptakes could be misinterpreted or read as being attributed to a malignant TNIC which in fact had no FDG accumulation. While observation of metabolic findings in conjunction with anatomical localization in PET/CT scans could prevent this misinterpretation, reading physicians in PET studies could have less false negative cases, and consequently, resulting in falsely higher sensitivity, NLR and NPV in this scenario. Moreover, in earlier PET alone studies, the definition of positive cases for FDG uptake was less strict. The descriptions such as “any uptake”^[13]^ or “clearly visible,”^[14]^ rather than “focal uptake,” which was more broadly used by PET/CT studies,^[6–8,11,26]^ could make this kind of misinterpretation happen more frequently. In this regard, we performed a subgroup analysis to include only studies with the description of “focal uptake” to define positive cases. However, the sensitivity, NLR and NPV of ^18^F-FDG-PET were still better than those of ^18^F-FDG-PET/CT in evaluating TNIC by Bethesda classification, albeit without statistical significance (Table 4). This indicates that although strict definition of positive cases could reduce the “superiority” of PET alone studies over PET/CT studies, misinterpretation of atypical FDG uptakes in the neck region might still exist and largely account for the overvaluation of the rule-out performance of ^18^F-FDG-PET in TNIC by Bethesda classification.

**Table 4:**
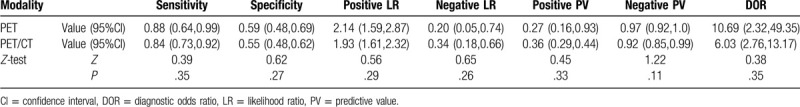
Subgroup analysis to include only studies with focal FDG uptake as definition of positive cases.

Although this comparison study was based on the most current evidence in the literature, it should be emphasized that the included studies exclusively focused on PET/CT or PET alone without a direct comparison between them in a specific population. Thus, our results might be undermined by differences in patient populations as well as different image interpretation protocols between the 2 comparison groups. In this regard, head-to-head comparisons in the same population are needed to reach a more powerful conclusion. Nonetheless, based on the results of the current study, we still advocate that future studies be performed with PET/CT rather than PET alone to avoid misinterpretation and overvaluation in diagnosing TNIC.

In our study, based on the Fagan nomogram we constructed, when the pre-test probability was set at 24%, the negative post-test probability could decrease to 12% for a PET/CT scan. This result supports that ^18^F-FDG-PET/CT holds moderate efficiency in ruling-out malignancy in this clinical setting. Moreover, as the goal of ruling out malignancy is to avoid unnecessary diagnostic surgeries on benign nodules, an ideal rule-out test should correctly identify as many benign ones as possible. In other words, apart from being efficient, an excellent rule-out test should also have a high benign diagnostic yield or high specificity to be cost-effective. For example, a recent health economic study on molecular testing in TNIC demonstrated that high specificity is critical to generate both positive health outcomes (fewer surgeries) and positive economic outputs (cost savings).^[30]^ In our meta-analysis, the pooled specificity for ^18^F-FDG-PET/CT was 0.56 (95% CI; 0.51–0.62). Based on our data, one unnecessary surgery could be avoided for every 2.3 PET/CT scan performed and 42% of the population tested would eventually avoid unnecessary surgical procedure and associated complications.

Nevertheless, the following limitations should be noted for the PET/CT results. Firstly, the inconsistency for the interpretation of PET images between studies is a major limitation of this study. In this meta-analysis, 5 PET/CT studies adopted visual analysis while the other 3^[5,9,27]^ did quantitative analysis. Besides, even for visual analysis, different studies used different definitions for a positive PET scan. In this regard, multicenter studies with unified image interpretation protocols should be performed in the future. Secondly, there is a large variation in the prevalence of malignancy for the PET/CT studies (range, 4%–50%). This might be attributed to the different epidemic rate in different regions as well as the different inclusion criteria of each study (Supplement Table 2, http://links.lww.com/MD/E309). Because the pre-test probability could significantly influence the predictive value of ^18^F-FDG- PET/CT, as is shown by the Fagan nomogram, the pooled PET/CT results in this meta-analysis should be interpreted with caution in an institution or a scenario with high prevalence of malignancy. Thirdly, there was moderate heterogeneity of sensitivity and specificity between PET/CT studies and this indicated a lack of consistency for the PET/CT results, although we did not identify source of heterogeneity. Finally, we admit that that the Bethesda system for thyroid cytology is evolving over the years but the definition of Bethesda III or IV nodules is slightly affected. Moreover, we included one PET/CT study which used the Italian consensus for cytological classification (ICCC) for evaluation of thyroid cytology,^[31]^ but the definition of ICCC III and IV nodules were similar to that of Bethesda III and IV nodules.

*In conclusion*, this meta-analysis reveals that in evaluating TNIC by Bethesda classification, the rule-out performance of ^18^F-FDG-PET is significantly better than ^18^F-FDG-PET/CT, although the latter represents a more objective and accurate technique. We hypothesize that the lack of precise localization of suspicious FDG uptake in the neck region may have contributed to this overvaluation for PET alone studies, and advocate that future studies be performed with PET/CT rather than PET alone to avoid misinterpretation and overvaluation in this scenario.

## Author contributions

**Conceptualization:** Wang Renjie, Zheng Xiuying.

**Data Collection:** Wan Qichang, Shen Jinming, Li Lu.

**Formal Analysis:** Wan Qichang, Shen Jinming.

**Methodology:** Shen Jinming.

**Software:** Wan Qichang.

**Writing – original draft:** Wang Renjie, Zheng Xiuying.

**Writing – review and editing:** Ji Bin.

## Supplementary Material

SUPPLEMENTARY MATERIAL
