# Supplementary material for: Comparison of 18F-FDG-PET and 18F-FDG-PET/CT for the diagnostic performance in thyroid nodules with indeterminate cytology: A meta-analysis
Source: Medicine (Baltimore). 2020 May 29;99(22):e20446. doi: 10.1097/MD.0000000000020446 (PMC12245225; doi:10.1097/MD.0000000000020446)
Supplement: SUPPLEMENTARY MATERIAL [file medi-99-e20446-s001.docx]

**Supplement Table 1 Meta-regression analysis for PET/CT studies**

| Covariate/Subgroup | No. of studies | Sensitivity (95% CI) | **P* value | Specificity (95% CI) | **P* value |
| --- | --- | --- | --- | --- | --- |
| Study design |  |  | 0.90 |  | 0.43 |
| Prospective  Retrospective | 5 | 0.78 (0.63,0.93) |  | 0.57 (0.50,0.63) |  |
|  | 2 | 0.78 (0.51,1.00) |  | 0.47 (0.36,0.57) |  |
| Data style |  |  | 0.57 |  | 0.09 |
| Patient-based  Lesion-based | 4 | 0.80 (0.62,0.98) |  | 0.52 (0.41,0.62) |  |
|  | 4 | 0.68 (0.39,0.96) |  | 0.65 (0.53,0.77) |  |
| Image analysis |  |  | 0.15 |  | 0.42 |
| Visual  Quantitative | 5 | 0.81 (0.64,0.97) |  | 0.55 (0.44,0.67) |  |
|  | 3 | 0.50 (0.04,0.97) |  | 0.61 (0.44,0.78) |  |
| Sample size |  |  | 0.64 |  | 0.14 |
| ≥ 50 | 5 | 0.78 (0.60, 0.95) |  | 0.54 (0.44, 0.64) |  |
| < 50 | 3 | 0.68 (0.32, 1.00) |  | 0.66 (0.51, 0.82) |  |

**CI, confidence interval.**

**Supplement Table 2 The inclusion, exclusion criteria of included studies**

|  | Year |  | Inconclusion criteria | Exclusion criteria |
| --- | --- | --- | --- | --- |
| 1. *Rosario et al.* | 2019 |  | 1. Nonautonomous TN 2. Nodule size> 1 cm 3. Cytology is indeterminate (Bethesda III or IV) 4. Not ‘highly suspicious’ on US | 1. With a history of familial thyroid carcinoma 2. exposed to radiation in childhood or adolescence 3. with a known diagnosis of thyroid carcinoma 4. with hypercalcitoninemia |
| 1. *Nguyen et al.* | 2018 |  | AUS/FLUS or FN/SFN | 1. B symptoms (e.g., weight, night sweats) 2. suspicious ultrasound examination (lymph node metastasis, suspicious thyroid tumor) 3. suspicious clinical examinations (e.g., recurrent laryngeal nerve palsy) 4. lymph node metastasis and/or distant metastasis on 18F-FDG PET/CT, histology other than of thyroid origin |
| 1. *Merten et al.* | 2017 |  | Suspicious for Hürthle cell neoplasm or follicular neoplasm | NA |
| 1. *Piccardo et al.* | 2016 |  | 1. Indeterminate cytology 2. Nodules size> 1 cm 3. TSH: 1–4 mIU/L 4. Undetectable thyroperoxidase   and thyroglobulin autoantibodies | NA |
| 1. *Pathak et al.* | 2016 |  | 1. Follicular or Hürthle cell neoplasms 2. Nodule size> 0.5 cm | NA |
| 1. *Muñoz Pérez et al.* | 2013 |  | 1. Follicular or Hürthle cell neoplasm 2. Euthyroid. | 1. Autonomous nodules 2. Contraindication for 18F-FDG PET/CT or FNA, 3. History or presence of another extrathyroidal cancer |
| 1. *Deandreis et al.* | 2012 |  | 1. AUS/FLUS and FN/SFN, 2. Nodule size> 1 cm 3. TSH: 0.5–4 mIU/L | 1. Diabetes mellitus 2. Abnormal TSH |
| 1. *Traugott et al.* | 2010 |  | 1. Adults with TN or dominant TN 2. Nodules palpable or >1 cm on Ultrasound 3. Scheduled for surgery 4. FNA: follicular lesion, Hürthle cell or oncocytic cell lesion, or atypical cytology | 1. Previous neck surgery 2. previous radiotherapy |
| 1. *Smith et al.* | 2008 |  | 1. Patients with a preoperative diagnosis of follicular neoplasm by FNA 2. Surgical excision has planned | NA |
| 1. *Hales et al.* | 2008 |  | All patients with TN: either follicular or Hürthle cell lesion by FNA | 1. Pregnancy 2. Breastfeeding 3. Previous neck surgery 4. >181 kg bodyweight |
| 1. *de Geus-Oei et al.* | 2006 |  | 1. Palpable TN 2. FNA: a follicular neoplasm or a Hürthle cell (oncocytic) neoplasm, if they showed atypical papillary cells, or if the sample was repeatedly insufficient 3. scheduled for hemithyroidectomy | 1. Diabetes mellitus 2. Pregnancy |
| 1. *Mitchell et al.* | 2005 |  | Microfollicular pattern | NA |
| 1. *Kresnik et al.* | 2003 |  | 1. All patients with TN 2. Hypoechogenic or no uptake on scintigraphy 3. Follicular or Hurthle cell proliferation on FNA 4. Scheduled for surgery | Autonomous goiter |

**AUS/FLUS: atypia of undetermined significance/follicular lesion of undetermined significance; FNA: fine-needle aspiration cytology; FN/SFN = follicular neoplasm/suspicious for a follicular neoplasm; NA = not available; TN: thyroid nodule; TSH: thyroid-stimulating hormone;**
